# ChromDMM: a Dirichlet-multinomial mixture model for clustering heterogeneous epigenetic data

**DOI:** 10.1093/bioinformatics/btac444

**Published:** 2022-07-04

**Authors:** Maria Osmala, Gökçen Eraslan, Harri Lähdesmäki

**Affiliations:** Department of Computer Science, Aalto University, Espoo 02150, Finland; Klarman Cell Observatory, Broad Institute of Harvard and MIT, Cambridge, MA 02142, USA; Department of Computer Science, Aalto University, Espoo 02150, Finland

## Abstract

**Motivation:**

Research on epigenetic modifications and other chromatin features at genomic regulatory elements elucidates essential biological mechanisms including the regulation of gene expression. Despite the growing number of epigenetic datasets, new tools are still needed to discover novel distinctive patterns of heterogeneous epigenetic signals at regulatory elements.

**Results:**

We introduce ChromDMM, a product Dirichlet-multinomial mixture model for clustering genomic regions that are characterized by multiple chromatin features. ChromDMM extends the mixture model framework by profile shifting and flipping that can probabilistically account for inaccuracies in the position and strand-orientation of the genomic regions. Owing to hyper-parameter optimization, ChromDMM can also regularize the smoothness of the epigenetic profiles across the consecutive genomic regions. With simulated data, we demonstrate that ChromDMM clusters, shifts and strand-orients the profiles more accurately than previous methods. With ENCODE data, we show that the clustering of enhancer regions in the human genome reveals distinct patterns in several chromatin features. We further validate the enhancer clusters by their enrichment for transcriptional regulatory factor binding sites.

**Availability and implementation:**

ChromDMM is implemented as an R package and is available at https://github.com/MariaOsmala/ChromDMM.

**Supplementary information:**

Supplementary data are available at *Bioinformatics* online.

## 1 Introduction

For over a decade, next-generation sequencing technologies have produced massive data amounts to quantify chromatin features, including nucleosomal histone modification locations, transcriptional regulatory factor (TRF) binding sites and chromatin accessibility (Boyle *et al.*, 2008; Mardis, 2007; Park, 2009). These chromatin-feature signals are routinely formed as counts of aligned sequencing reads at consecutive non-overlapping genomic windows (or bins) along the entire genome or a short DNA stretch. These coverage signals at regulatory elements, such as enhancers, are often investigated to understand the underlying biological mechanisms in the regulation of gene expression. Moreover, the signals can be visualized as heatmaps by aligning them within a genomic window centred at the loci (see Fig. 4 as an example). The average *aggregate patterns* of the coverage signals, illustrated on top of the heatmaps, reveal the positional correlations and recurrent patterns in the signals. However, the set of analysed genomic regions can be biologically heterogeneous; in other words, it consists of multiple unknown subclasses. Therefore, the aggregate plot derived from all regions falsely displays the superposition of several different chromatin signatures. Consequently, we need a clustering method to reveal the subclasses.

The clustering method must consider the following properties of the chromatin-feature data. First, the data are heterogeneous containing sparse count data as well as varying coverage intensities and patterns. Second, the anchor positions of regulatory elements are typically uncertain; genomic regions need shifting, that is, the coverage signals need alignment with respect to each other to refine the aggregate patterns. Third, chromatin features can be asymmetric concerning the anchor points due to directional biomolecular mechanisms, such as transcription. Therefore, the coverage signals need strand-orientation (flipping).

Several methods have been proposed for the epigenetic data clustering, such as hierarchical clustering (Kundaje *et al.*, 2012; Nielsen *et al.*, 2012) and *k*-means (Groux and Bucher, 2019; Heintzman *et al.*, 2007; Ye *et al.*, 2011). The hierarchical clustering tool CAGT by Kundaje *et al.* (2012) groups chromatin profiles at functional genomic elements into clusters using *k*-medians algorithm. This procedure is followed by merging redundant clusters through the hierarchical agglomerative clustering utilizing either correlation or Euclidean distance. CAGT implements profile flipping but no shifting. Some clustering methods such as ChromaSig (Hon *et al.*, 2008), CATCHprofiles (Nielsen *et al.*, 2012) and ChExMix (Yamada *et al.*, 2019) examine the chromatin-feature enrichment in the entire genome instead of clustering predefined sets of genomic elements. ChromaSig (Hon *et al.*, 2008) is a clustering method that implements both shifting and flipping, and it assumes that the read counts are normally distributed. CATCHprofiles (Nielsen *et al.*, 2012) is another hierarchical clustering approach combined with pairwise alignment. CATCHprofiles merges, aligns and orients the most similar profile pairs to remaining profiles iteratively based on correlation or Euclidean distance. This results in a very exhaustive search. ChExMix (Yamada *et al.*, 2019) is designed to cluster the ChIP-seq or the higher resolution ChIP-exo (Rhee and Pugh, 2011) and ChIP-nexus (He *et al.*, 2015) read count footprints together with the DNA sequence information at the TRF binding sites. The footprints and DNA motifs are not equal at all binding sites, for example, due to the TRF of interest interacting with distinct sets of other regulatory proteins. ChExMix models read counts as being generated by a mixture of binding events and their subtypes along the entire genome. The model is formulated as a probabilistic mixture model with multinomial component distributions and assuming sparsity-inducing Dirichlet priors on the mixture weights and binding event subtypes. The multinomial parameters, that is, the binding event positions, are assumed to follow a Bernoulli distribution. The model parameters are estimated using expectation–maximization (EM) algorithm, and as a result one obtains the responsibility of each binding subtype at each binding event in generating each sequenced read. ChExMix also considers the orientation of the footprints and allows small shifting.

A probabilistic mixture model designed to cluster chromatin-feature signals at regulatory elements was introduced by Nair *et al.* (2014). The model is denoted as ChIP-partitioning and it considers the above-mentioned requirements for the chromatin-feature clustering method. ChIP-partitioning models the statistical variation in the coverage signals using independent Poisson distributions. Nair *et al.* (2014) demonstrated that ChIP-partitioning outperforms the hierarchical and *k*-means clustering methods, particularly when clustering low-coverage count data. However, next-generation sequencing data are typically overdispersed; the data variation is larger than expected by the Poisson distribution. Therefore, many overdispersed models have been proposed, for example, for RNA-seq data analysis (Robinson *et al.*, 2010). Moreover, previous studies on clustering chromatin features do not provide rigorous probabilistic methods for clustering multiple chromatin features simultaneously. The previous methods also lack a principled method for determining the unknown number of clusters.

We propose a probabilistic clustering method ChromDMM that exploits the discrete, sparse, heterogeneous and overdispersed nature of the sequencing data. ChromDMM builds on the mixture of Dirichlet-multinomial compound distributions originally proposed for clustering microbial data (Holmes *et al.*, 2012). We extend the model to account for the presence of multiple epigenetic coverage signals at the same genomic locus, so that each mixture component exhibits a set of Dirichlet-multinomial compound distributions. We also extend the model with the profile shifting and flipping features that can probabilistically account for the inaccuracies in the positions and strand-orientations of the clustered genomic elements. In addition, owing to the regularization of the mixture component parameters, ChromDMM can smooth the chromatin-feature patterns at successive bins along the genomic regions. Finally, our probabilistic model can naturally utilize the well-known model selection methods to determine the optimal number of clusters. The following Section 2 presents the ChromDMM model and its inference in detail. Section 3 analyses the performance of ChromDMM on simulated and real chromatin-feature data and compares its performance against ChIP-partitioning (Nair *et al.*, 2014) and SPar-K (Groux and Bucher, 2019).

## 2 Materials and methods

The data for a chromatin feature across *N* genomic loci is represented as a *N *×* L* matrix $X={\left[x_{1},\ldots ,x_{N}\right]}^{T}$, where $x_{i}={\left[x_{i1},\ldots ,x_{iL}\right]}^{T}$ denotes the data for the *i*th genomic window. The length of $x_{i}$ is defined by the size of the genomic locations *W* and resolution *B* as L=W/B. For example, data extracted in *W *=* *2000 base pair (bp) windows centred at the anchor points with the resolution *B *=* *40 bps results in coverage signals of length *L *=* *50. The element *x_ij_* denotes the number of sequencing reads whose starting position (5′ end) is aligned to bin *j* of locus *i*. To be exact, *x_ij_* denotes, for example, the histone modification ChIP-seq read counts minus the sequencing-depth normalized control counts (see Supplementary Section S4.2). Collectively, the data for *M* chromatin features are represented as a *N* × *ML* matrix $X^{*}=[X^{\left(1\right)},X^{\left(2\right)},\ldots ,X^{\left(M\right)}]={\left[x_{1}^{*},\ldots ,x_{N}^{*}\right]}^{T}$, where $x_{i}^{*}={\left[x_{i}^{\left(1\right)}{}^{T},\ldots ,x_{i}^{\left(M\right)}{}^{T}\right]}^{T}$ denotes a vector of length *ML* that contains the *M* chromatin feature vectors of a single genomic locus *i*.

### 2.1 Product Dirichlet-multinomial mixture model

The read counts **x** across the *L* bins are naturally modelled by the multinomial distribution with parameters $p={\left[p_{1},\ldots ,p_{L}\right]}^{T}$ ($\sum_{j=1}^{L}p_{j}=1$). We further assume the multinomial parameters **p** are distributed according to a conjugate Dirichlet distribution with hyperparameters α. Marginalizing out the multinomial parameters from the joint distribution of **x** and **p** results in an overdispersed Dirichlet-multinomial compound distribution parameterized by α. The Dirichlet-multinomial distributions can be utilized as the component distributions in a mixture model for the probabilistic clustering.

Compared with the standard Dirichlet-multinomial mixture model (Holmes *et al.*, 2012), we implement two extensions. First, we assume that the likelihood of $x^{*}$ is a product multinomial distribution, each multinomial with the chromatin-feature-specific parameters $p^{\left(m\right)}$. This enables modelling several chromatin features simultaneously. Second, we assume the parameters $p^{*}=(p^{\left(1\right)},\ldots ,p^{\left(M\right)})$ to have a mixture prior with *K* mixture components; each component *k* is a product of *M* Dirichlet distributions again with the chromatin-feature-specific hyperparameters $\alpha_{k}=[\alpha_{k}^{\left(1\right)},\ldots ,\alpha_{k}^{\left(M\right)}]$. Let the parameters of the product-Dirichlet mixture for all *K* mixture components and *M* chromatin features be represented as a *L* × *KM* matrix $\alpha^{*}=\left[\alpha_{1}^{\left(1\right)},\ldots ,\alpha_{K}^{\left(1\right)},\ldots ,\alpha_{1}^{\left(M\right)},\ldots ,\alpha_{K}^{\left(M\right)}\right]$. The mixture prior for $p^{*}$ is

$$p(p^{*}|\alpha^{*},\pi )=\sum_{k=1}^{K}\pi_{k}\prod_{m=1}^{M}\text{Dirichlet}\;(p^{\left(m\right)}|\alpha_{k}^{\left(m\right)}),$$

where $\pi =(\pi_{1},\ldots ,\pi_{K})$ denotes the mixture weights. Holmes *et al.* (2012) showed that compounding the multinomial distribution with the Dirichlet mixture prior results in an analytically tractable likelihood. Similarly, in the case of the product-multinomial with the product-Dirichlet mixture prior, the parameters of the product-multinomial can also be marginalized analytically to derive a closed-form expression for the likelihood of $X^{*}$ as (see Supplementary Section S1.4 for a detailed derivation)

(1) $$p(X^{*}|\alpha^{*},\pi )=\prod_{i=1}^{N}\sum_{k=1}^{K}\pi_{k}\prod_{m=1}^{M}\;\text{Dirichlet-multinomial}\;(x_{i}^{\left(m\right)}|\alpha_{k}^{\left(m\right)}).$$

Instead of seeking to obtain the maximum-likelihood estimates for the model parameters, we adopt the Bayesian approach by introducing a prior distribution for the component parameters $\alpha^{*}$.

To account for the correlations between the (expected) read counts at consecutive bins along the chromatin signal, we define a regularized Gamma hyperprior for the mixture component parameters $\alpha^{*}$ as

(2) $$p(\alpha^{*})\propto \prod_{m=1}^{M}\prod_{k=1}^{K}\Gamma (h_{k}^{\left(m\right)}|\eta_{h},\nu_{h})\prod_{j=1}^{L}\Gamma (\alpha_{kj}^{\left(m\right)}|\eta ,\nu ),$$

where all $\alpha_{kj}^{\left(m\right)}$ have their own independent Gamma prior with fixed shape *η* and rate *ν* parameters, and the regularization terms

(3) $$h_{k}^{\left(m\right)}=\sum_{j=2}^{L}{\left(\alpha_{kj}^{\left(m\right)}-\alpha_{k,\;j-1}^{\left(m\right)}\right)}^{2}$$

also have their own independent Gamma prior with shape *η_h_* and rate *ν_h_* parameters. Inclusion of the regulatory terms in the prior favours smooth mixture component parameters. A more detailed expression of the proportional distribution of the prior $p(\alpha^{*})$ is shown in Supplementary Equation (S9) and an example of the effect of the regularization is demonstrated in Supplementary Section S1.6.

Mixture models involve the latent cluster membership variables **z**; each observed $x_{i}^{*}$ is associated with a corresponding unobserved categorical latent variable $z_{i}$. The variable $z_{i}={\left(z_{i1},\ldots ,z_{iK}\right)}^{T}$ is a *K*-dimensional indicator vector: if sample *i* originates from cluster *k*, *z_ik_* = 1; otherwise *z_ik_* = 0. The variables $z_{i}$ are collected in a *N *×* K* matrix $Z={\left[z_{1},\ldots ,z_{N}\right]}^{T}$. The proposed model is parameterized by $\theta =(\alpha^{*},\pi )$ and is presented as a directed acyclic graph in Supplementary Fig. S1 together with the distributions of individual components.

### 2.2 The EM algorithm

The posterior $\text{log}\;p(X^{*}|\theta )+\text{log}\;p(\theta )$ cannot be maximized directly. Instead, the MAP estimates for θ and the probabilistic cluster assignments are obtained by an iterative approach, the EM algorithm (Bishop, 2006). For the derivation of the EM algorithm assume a distribution for **Z**, q(Z). Then, the Jensen’s inequality provides a lower bound for the posterior distribution

$$\;\text{log}\;p(\theta^{*}|X^{*})\geq E_{q(Z)}[\text{log}\;p(\theta ,Z|X^{*})]=E_{q(Z)}[\text{log}\;p(X^{*},Z|\theta )]+p(\theta )+\text{constant},$$

where $\text{log}\;p(X^{*},Z|\theta )$ is the complete data log-likelihood and the constant term is independent on θ. Assuming some initial estimates for the parameters $\theta^{\text{old}}$ and defining $q(Z)=p(Z|X^{*},\theta^{\text{old}})$, the lower bound (without the constant term) is

$$Q(\theta ,\theta^{\text{old}})=E_{p(Z|X^{*},\theta^{\text{old}})}\left[\text{log}\;p(X^{*},Z|\theta )\right]+\text{log}\;p(\theta )=\sum_{i=1}^{N}\sum_{k=1}^{K}E[z_{ik}]\sum_{m=1}^{M}\;\text{log}\;p(x_{i}^{\left(m\right)}|\theta )+\sum_{i=1}^{N}\sum_{k=1}^{K}E[z_{ik}]\;\text{log}\;\pi_{k}+\text{log}\;p(\alpha^{*})+\text{log}\;p(\pi ),$$

where the expectation is wrt the posterior probabilities of the cluster assignments conditional on the current parameter estimates $\theta^{\text{old}}$, that is, $E[z_{ik}]=p(z_{ik}=1|x_{i}^{*},\theta^{\text{old}})$. The likelihood term $p(x_{i}^{\left(m\right)}|\theta )$ is the Dirichlet-multinomial compound distribution for the *m*th chromatin feature. For a detailed derivation, see Supplementary Section S1.8.

Algorithm 1: EM algorithm for ChromDMM

 **Input**: Data $X^{*}$ for all *M* chromatin features, the number of clusters *K*, hyper-parameters $\left(\eta ,\nu ,\eta_{h},\nu_{h}\right)$

 **Output**: MAP estimates $\hat{\theta }$ and $p(Z|X,\hat{\theta })$

**1** Parameters $\theta =(\lambda_{1}^{\left(1\right)},\ldots ,\lambda_{K}^{\left(1\right)},\ldots ,\lambda_{1}^{\left(M\right)}\ldots ,\lambda_{K}^{\left(M\right)},\pi )$;

**2 /**/Initialisation to obtain  $\theta^{\text{old}}$

**3** Initialise $E[z_{ik}]$ using soft *k*-means on concatenated data;

**4** Initialise $\lambda_{jk}^{\left(m\right)}$ by $\arg\max_{\lambda^{*}}Q(\theta ,\theta^{\text{old}})$ using BFGS;

**5 /**/The EM algorithm loop

**6 while** *the lower bound* $Q(\theta ,\theta^{\text{old}})$  *not converged* **do**

**7**  **/**/E-step:

**8**  Compute $p(Z|X,\theta^{\text{old}})$, i.e., $E(z_{ik})$;

**9**  **/**/M-step:

**10**  $\lambda^{\left(*,\text{new}\right)}=\text{arg}{\text{max}}_{\lambda^{*}}Q(\theta ,\theta^{\text{old}})$ using BFGS

**11**  Update mixing weights π: $\pi_{k}^{\text{new}}=\frac{1}{N}\sum_{i=1}^{N}E[z_{ik}]$

**12**  $\theta =\left(\lambda^{\left(*\;,\text{new}\right)},\pi^{\text{new}}\right)$

**13 end**

In the EM algorithm, E-steps and an M-steps are repeated, until convergence of the lower bound $Q(\theta ,\theta^{\text{old}})$. In the E-step, the posterior probability that a sample *i* belongs to a cluster *k* given the current parameter estimates $\theta^{\text{old}}$ is obtained using the standard Bayes rule as

$$p(z_{ik}=1|x_{i}^{*},\theta^{\text{old}})=\frac{p(z_{ik}=1|\theta^{\text{old}})p(x_{i}^{*}|z_{ik}=1,\theta^{\text{old}})}{\sum_{k\prime =1}^{K}p(z_{ik\prime }=1|\theta^{\text{old}})p(x_{i}^{*}|z_{ik\prime }=1,\theta^{\text{old}})},$$

where $p(x_{i}^{*}|z_{ik}=1,\theta^{\text{old}})=\prod_{m=1}^{M}p(x_{i}^{\left(m\right)}|z_{ik}=1,\theta^{\text{old}})$ is the likelihood of the sample *i* conditioned with cluster *k*, that is, the product Dirichlet-multinomial distribution. The term $p(z_{ik}=1|\theta^{\text{old}})$ corresponds to the mixture weight *π_k_*. In the M-step, as a closed-form solution of $\alpha^{*}$ that maximizes $Q(\theta ,\theta^{\text{old}})$ is unattainable, the lower bound is maximized wrt $\alpha^{*}$ using Broyden–Fletcher–Goldfarb–Shanno (BFGS) method provided in R (Broyden, 1970). In addition, the component parameters $\alpha_{kj}^{\left(m\right)}$ are constrained to be positive by a reparameterization $\lambda_{k}^{\left(m\right)}=\text{log}\;\alpha_{k}^{m}$ and by re-defining the prior for $\lambda^{*}$ accordingly using the multivariate change of variables method. For more details on deriving the equations for the model inference, see Supplementary Sections S1.7–S1.15. The steps of the EM algorithm are summarized in Algorithm 1. In the initilization, the cluster membership probabilities $E\left[z_{ik}\right]$ are obtained with the soft *k*-means clustering (MacKay, 2003) on concatenated chromatin features (see details in Supplementary Section S4.1). For a given number of clusters, the EM algorithm is run multiple times each with different random initialization. Note that it is trivial to parallelize the computation across the multiple runs as well as across varying numbers of clusters.

### 2.3 Chromatin feature profile shifting and flipping

We extend the product Dirichlet-multinomial mixture model with shifting and flipping features. For profile shifting, we first define the maximum amount of shifting, for example, 400 bp, both upstream and downstream. With a given bin size (e.g. B=40bp), this results in $S=\frac{2\times 400\text{bp}}{40\text{bp}}+1=21$ possible shift states, where the shift state $s=\frac{S+1}{2}$ corresponds to no shift. In addition, the length of the Dirichlet parameters $\alpha_{k}^{\left(m\right)}$ is extended from *L* to L+S−1. When evaluating the likelihood model from Equation (1) for a shift state *s*, we use the corresponding *L*-length subset of the extended Dirichlet parameters for each mixture component *k*, denoted as $\alpha_{ks}^{*}=(\alpha_{k,s}^{*},\alpha_{k,s+1}^{*},\ldots ,\alpha_{k,s+L-1}^{*})$. For profile flipping, we either compute the likelihood model definition with a shift state *s* (using again the *L*-length subset of the Dirichlet parameters) if *f *=* *1, or reverse the order of the Dirichlet parameters if *f *=* *2. Formally, we denote the shifting and flipping-aware likelihood model for a single genomic locus as $p(x^{*}|\alpha_{sf}^{*},\pi )$.

For each locus, we can define prior probabilities for the shift and flip states. The prior shift state probabilities for the genomic locus *i* are denoted as $\xi_{i}=(\xi_{i1},\ldots ,\xi_{iS})$, where $\sum_{s=1}^{S}\xi_{is}=1$. If the genomic loci and their anchor points are defined using ChIP-seq summits, then the prior for shift states can be defined, for example, as a pyramid-shaped prior that has the highest probability at the ChIP-seq peak summit (corresponding to no-shift state) and linearly decreasing the prior to zero beyond the maximum shift state. Similarly, we can define prior flip state probabilities $\zeta_{i}$ for each locus *i*, where $\zeta_{i1}+\zeta_{i2}=1$.

In ChromDMM with the shifting and flipping features, the latent cluster membership variables are re-defined as follows: *z_iksf_* = 1 if the sample *i* originates from the cluster *k*, has shift state *s* and has strand-orientation *f*; otherwise, *z_iksf_* = 0. These latent variables are stored in N×K×S×2 matrix (or tensor) **Z**. We show in Supplementary Section S2.3 that the EM algorithm can be derived similarly as in Section 2.2, resulting in the following lower bound for the posterior distribution of parameters θ
 

$$\begin{array}{l} Q(\theta ,\theta^{\text{old}})=\sum_{k=1}^{K}\;\text{log}\;\pi_{k}\sum_{i=1}^{N}\sum_{s=1}^{S}\sum_{f=1}^{2}E[z_{\mathrm{iksf}}]\\ +\sum_{i=1}^{N}\sum_{s=1}^{S}\;\text{log}\;\xi_{is}\sum_{k=1}^{K}\sum_{f=1}^{2}E[z_{\mathrm{iksf}}]\\ +\sum_{i=1}^{N}\sum_{f=1}^{2}\;\text{log}\;\zeta_{if}\sum_{k=1}^{K}\sum_{s=1}^{S}E[z_{\mathrm{iksf}}]\\ +\sum_{i=1}^{N}\sum_{k=1}^{K}\sum_{s=1}^{S}\sum_{f=1}^{2}E[z_{\mathrm{iksf}}]\sum_{m=1}^{M}\;\text{log}\;p(x_{i}^{\left(m\right)}|\alpha_{\mathrm{ksf}}^{\left(m\right)})\\ +\text{log}\;p(\theta ), \end{array}$$

where $E[z_{\mathrm{iksf}}]=p(z_{\mathrm{iksf}}=1|x_{i}^{*},\theta^{\text{old}})$. Note that the above mixture model can be applied (i) only with shifting, (ii) only with flipping or (iii) with both shifting and flipping. In the case of (i), we simply drop the index *f* and the corresponding sums and in the case of (ii), we simply drop the index *s* and the corresponding sums. For more details on the derivations of equations needed to infer the shifting and flipping-aware model, see Supplementary Section S2.

After learning the model parameters with the EM algorithm, we infer the final cluster assignment ${\tilde{k}}_{i}$ for each sample *i* by marginalizing the shift and flip states. Similarly, we choose the final flip and shift states, ${\tilde{f}}_{i},{\tilde{s}}_{i}$, that maximize the posterior given the optimal cluster ${\tilde{k}}_{i}$ by marginalizing the shift and flip states, respectively,

(4) $${\tilde{k}}_{i}=\underset{k}{\text{arg}\text{max}}\sum_{s=1}^{S}\sum_{f=1}^{2}p(z_{\mathrm{iksf}}=1|x_{i}^{*}),$$

 

(5) $${\tilde{f}}_{i}=\underset{f}{\text{arg}\text{max}}\sum_{s=1}^{S}p(z_{i{\tilde{k}}_{i}sf}=1|x_{i}^{*}),$$

 

(6) $${\tilde{s}}_{i}=\underset{s}{\text{arg}\text{max}}\sum_{f=1}^{2}p(z_{i{\tilde{k}}_{i}sf}=1|x_{i}^{*}).$$

### 2.4 Choosing the number of clusters and identifiability aspects

For probabilistic clustering methods, the Bayesian model selection is commonly used to guide the selection of an appropriate number of clusters *K*. While the exact computation of the marginal likelihood is impractical, we can directly apply the commonly used approximative methods, such as the Bayesian information criterion (BIC) (Schwarz, 1978) or the Akaike information criterion (AIC) (Akaike, 1973).

There are inherent unidentifiability issues in ChromDMM results. Firstly, as in any clustering method, the inferred cluster labels can be switched between two clusters without affecting the clustering accuracy. Secondly, unless informative prior for strand-orientation is provided, the flip state indexes (1 or 2) can always be reversed. For biological interpretation, the aligned and flipped profiles need to be visualized after clustering and compared with the underlying directionality of the genomic region, such as direction of transcription 3′ → 5′ or 5′ → 3′, if known. The learned shift state is also affected by the learned flip state. While evaluating the performance of ChromDMM and other methods on simulated data, we consider these aspects.

## 3 Results

### 3.1 Clustering simulated data

#### 3.1.1 Data simulation and choice for hyperparameters

We used simulated data to investigate the clustering accuracy of ChromDMM, ChIP-Partitioning and SPar-K when the data contain varying number of chromatin features and varying read coverages. For comparison, we repeated some experiments on the simulated data presented by Nair *et al.* (2014). We simulated data containing two clusters using the R-code presented in Nair *et al.* (2014) (see their Supplementary Material, page 13). We generated 1000 samples per cluster using low-coverage parameter values *f *=* *0.5 and *f *=* *1. We found that at these low-coverage parameter values, most of the simulated profiles were zero vectors. Thus, we first generated 10 000 samples for both clusters and randomly selected 1000 non-zero vectors for both clusters. The data generation was repeated 100 times. An example of simulated dataset is presented in Supplementary Fig. S8. The cluster-wise aggregate profiles are Gaussian-shaped with varying location of the mean and variance.

To generate more realistic simulated data, we first clustered data for four chromatin features (H3K4me1, H3K27ac, RNA polymerase II and MNase-seq) at 1000 enhancers from the ENCODE project (The ENCODE Project Consortium, 2012) by ChromDMM requiring the inference of both the shift and flip states. For more details, see Supplementary Methods S4.2. From the fitted model, we chose the Dirichlet parameters $\alpha_{k}^{\left(m\right)}$ for two clusters. These parameters were used to sample the multinomial parameters $p_{i}^{\left(m\right)}$ and finally the profiles $x_{i}^{\left(m\right)}$ by varying the chromatin-feature-specific coverage between 10, 20, 50 and 100. For each experiment, we simulated 100 datasets. We used the area under receiver operations characteristics (AUC) curve as the performance measure for the clustering accuracy. For more details and the visualization of a simulated dataset with a coverage of 100, see Supplementary Section S4.4.

We performed hyperparameter sweeps on the simulated data to determine robust default values for the ChromDMM model. Briefly, for the Gamma prior for the Dirichlet parameters $\alpha_{kj}^{\left(m\right)}$ (parameterized by hyperparameters *η* and *ν*), we observed that the results were not sensitive to hyperparameter values and conclude that the choice of η=1.1 and ν=0.1 results in a good performance (Supplementary Fig. S5). We also performed the prior predictive checks using the ancestral sampling of the data from prior hyperparameters and demonstrated that the amount of variation generated from the prior is comparable to the variation in the real data (Supplementary Fig. S6). Similarly, we chose the hyperparameter values for the regularization term $h_{k}^{m}$. The Gamma prior with mean 1 and variance 0.1 corresponding to hyperparameters $\eta_{h}=\nu_{h}=10$ resulted in a robust clustering performance (see Supplementary Fig. S7 and Supplementary Section S4.5 for more details). We set the above hyperparameter values as defaults, but a user can, for example, perform prior predictive checks for his/her data and adjust the hyperparameters, if necessary.

We investigated the ability of AIC and BIC to choose the correct number of clusters (two) for the simulated data. We fitted ChromDMM with varying the number of clusters (from 1 to 3). The proportions of cluster numbers selected by AIC and BIC in 100 simulated datasets are presented in Supplementary Fig. S16a for 1000 samples and in Supplementary Fig. S16b for 6000 samples. We conclude that AIC and BIC detect the correct number of clusters more reliably when the coverage of the chromatin modifications and/or the number of samples increases, although BIC tends to underestimate the number of clusters. The computation times of the three methods (with default parameters) to cluster simulated data containing two clusters and two chromatin features both with coverage 100 were: half an hour (ChromDMM), ca. 10 min (ChIP-Partitioning) and seconds (SPar-K).

#### 3.1.2 ChromDMM infers accurate clusters

**Fig. 1.**
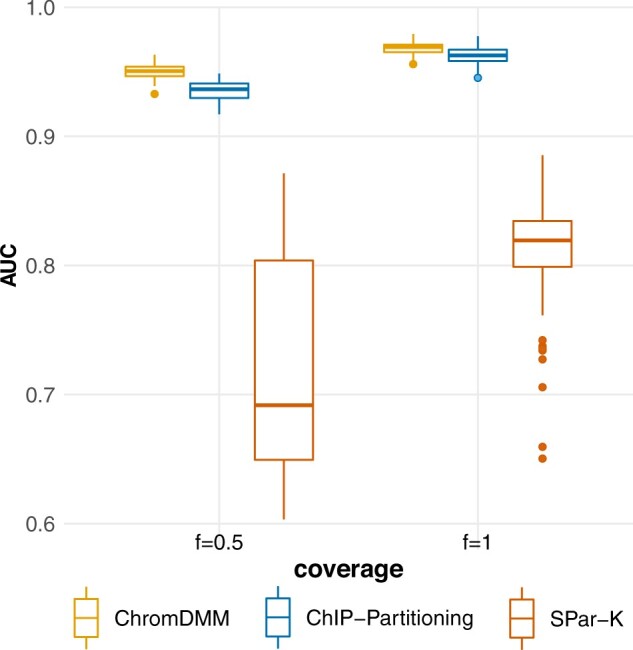
AUC values for clustering simulated data that were generated as in Nair *et al.* (2014) and contain two clusters and one chromatin feature. The chromatin feature coverage parameter *f* was varied between 0.5 and 1. Boxplots represent results for 100 datasets

We compared ChrommDMM against ChIP-partitioning and SPar-K (both applied with the default parameters) in clustering simulated data that were generated as in Nair *et al.* (2014). The clustering performance of ChromDMM exceeded the performance of ChIP-partitioning and SPar-K (Fig. 1). Similarly as in Nair *et al.* (2014), we also report the Pearson correlation coefficients between the true cluster-wise aggregate patterns and the inferred aggregate patterns (Supplementary Fig. S9). In general, the correlations are similar to values obtained by Nair *et al.* (2014). For the first cluster, the correlations obtained by ChromDMM are lower than for ChIP-partitioning, whereas for the second cluster, the correlations obtained by ChromDMM are slightly higher.

**Fig. 2.**
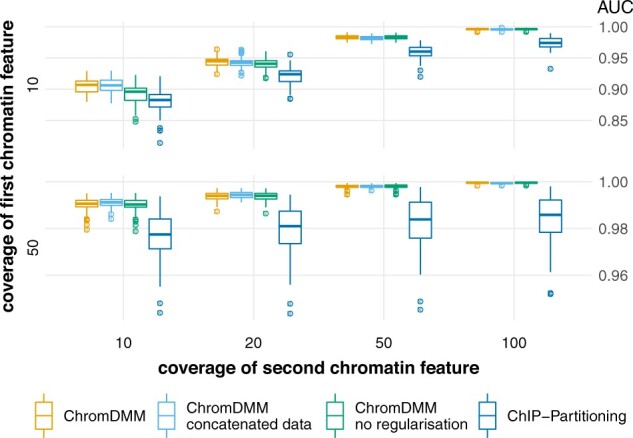
AUC values for clustering simulated datasets that contain two clusters and two chromatin features (H3K4me1 and RNA POL II). The chromatin feature coverages were varied between 10, 20, 50 and 100 for RNA POL II, and between 10 and 50 for H3K4me1 (see Supplementary Fig. S10 for more combinations). Boxplots represent results for 100 datasets

We compared ChrommDMM against ChIP-partitioning and SPar-K in clustering more realistic simulated data that contained two clusters and two chromatin features (H3K4me1 and RNA POL II). The clustering performance of ChromDMM exceeded the performance of ChIP-Partitioning (Fig. 2 and Supplementary Fig. S10). SPar-K performed poorly in these comparisons, particularly when the coverages were low (Supplementary Fig. S11). Similar results were obtained on data containing only a single feature (Supplementary Fig. S12). For comparison, Figure 2 and Supplementary Fig. S10 present also results for an experiment where ChromDMM was fitted either on concatenated chromatin profile data or without the regularization term. The regularization improved the clustering performance especially when the coverage for the first chromatin feature (H3K4me1) was low, whereas the use of non-concatenated chromatin profile data resulted in only a marginal improvement in this simulation setting.

#### 3.1.3 Clustering accuracy improves with the number of features

We experimented with the number of chromatin features; beginning from a single feature (H3K4me1), the number of features was increased to four by adding RNA Pol II, H3K27ac and MNase-seq. Supplementary Fig. S13a shows how the clustering accuracy increases together with the number of chromatin features for a signal coverage of 10. Supplementary Fig. S13b presents similar results for a varying coverage, where the coverage of the first set of chromatin features was 10 (H3K4m1, H3K27ac) and the coverage of the second set of chromatin features was 50 (RNA Pol II, MNase-seq). We conclude that for ChromDMM and ChIP-partitioning, the clustering performance increases as a function of the number of chromatin features, whereas for SPar-K the improvement is less consistent. Regardless of the number of chromatin features, ChromDMM obtains the best performance.

#### 3.1.4 ChromDMM infers accurate shift and flip states

The simulated data were also artificially shifted and flipped as described in Supplementary Section S4.4. Briefly, the random shifts were constrained to be multiple of the data resolution (*B *=* *40 bp) and drawn from the Skellam distribution with mean zero and a variance that included the randomly sampled shifts between –400 and +400 bp. Similarly, the flip states were sampled randomly with equal probability for both strand-orientations. For more details, see Supplementary Algorithm S3.

**Fig. 3.**
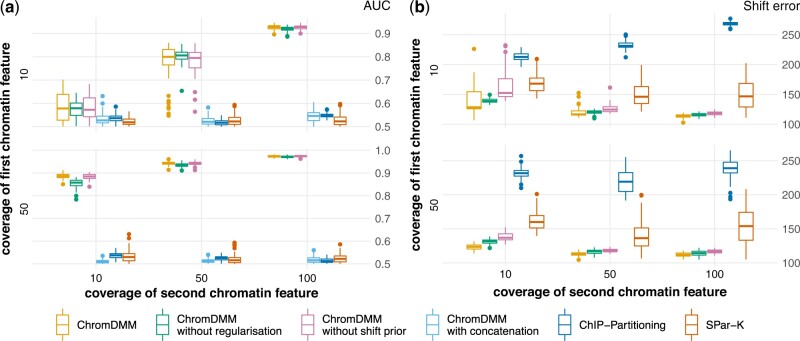
AUC values (a) and average shift errors in bps (b) for clustering simulated data that contain two clusters, two chromatin features and randomly sampled shift and flip states. The chromatin feature coverages varied between 10, 50 and 100. Results are shown for ChromDMM, ChIP-partitioning and SPar-K. For comparison, ChromDMM was applied also on concatenated chromatin features. ChromDMM was inferred also with the uniform shift state prior and without the regularization term. Boxplots represent results for 100 datasets

The clustering accuracy of ChromDMM, ChIP-partitioning and SPar-K was demonstrated on the randomly shifted and flipped simulated data. We experimented with four versions of ChromDMM: (i) ChromDMM with the regularization term and with the pyramid-shaped shift state prior, (ii) same as (i) but with concatenated chromatin features, (iii) ChromDMM without the regularization term and with the pyramid-shaped shift prior and (iv) ChromDMM with a uniform prior for the shift states and with the regularization term. The clustering accuracies of the methods are presented in Fig. 3a. Methods considering the concatenated chromatin features, including ChIP-partitioning and SPar-K, performed poorly in these comparisons and notably they failed to improve their performance while increasing the coverage values. ChromDMM outperformed other methods and its clustering performance was further improved by both the informative shift state prior and the regularization, particularly when the coverage of either chromatin feature was low.

The methods were compared with their accuracy to correctly infer the shift and the flip states of the genomic regions. ChromDMM and ChIP-partitioning infer the most probable shift and flip states for each sample *i* from the latent variable probabilities shown in Equations (5) and (6), respectively, whereas SPar-K outputs the inferred shift and flip states separately. The flip state error was defined as the proportion of incorrectly inferred flip states in a given experiment (recall the identifiability aspects from Section 2.4). Similarly, the shift error for each sample was computed as the absolute difference between the true shift and the inferred shift in nucleotides. The average shift error over all *N* samples was reported as the final shift error for a single experiment.

The flip errors for the simulated shifted and flipped data containing two chromatin features and two clusters are presented in Supplementary Fig. S14. On average, the flip errors decreased as the coverages increased and they were lower for the ChromDMM methods compared with ChIP-partitioning and SPar-K. The flip errors were only slightly affected by whether the ChromDMM fit was inferred without the shift prior or without the regularization. The resulting shift errors for the simulated data are shown in Fig. 3b. Again, the average shift errors decreased as the coverages increased and the shift errors were lowest for the ChromDMM methods. The shift state inference of ChromDMM was further improved by the informative shift prior and the regularization of the mixture component parameters $\alpha^{*}$. In contrast to the other methods, the shift errors for ChIP-partitioning remained high even with large coverage values. This likely results from the cluster-assigned patterns drifting from the profile centre positions, that is, ChIP-partition selects a profile whose unimodal peak or valley between the two-modal peak is shifted far from the profile centre and aligns the rest of the profiles according to this single profile (Supplementary Fig. S15d). The cluster patterns inferred by SPar-K also drift (Supplementary Fig. S15e), whereas ChromDMM centres the peaks and valleys to the profile centres (Supplementary Fig. S15c). This desirable behaviour of ChromDMM stems partly from the robustness of the probabilistic treatment and the shift state prior.

Finally, we investigated the ability of AIC and BIC to choose the correct number of clusters (two) in the simulated shifted and flipped data (Supplementary Fig. S17). In contrast to the simpler model studied in Section 3.1, the more complex ChromDMM model with a high number of parameters is heavily penalized by AIC and BIC, and thus require higher coverage and larger number of samples to detect the correct number of clusters.

### 3.2 Clustering enhancers in ENCODE data

#### 3.2.1 ChromDMM reveals distinctive enhancer clusters

**Fig. 4.**
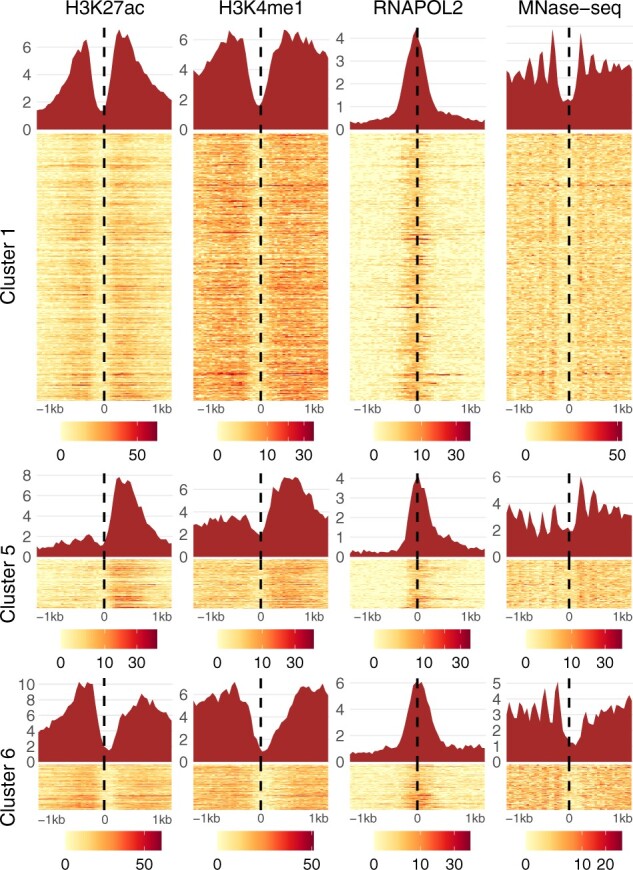
Enhancer clusters revealed by ChromDMM. The coverage signals of individual enhancers assigned to the clusters are visualized as heatmaps. The aggregate patterns are visualized on top of the heatmaps. Four of the 10 chromatin features for three of the six clusters are shown. The full set of clusters and chromatin features resulting from enhancer clustering by ChromDMM are presented in Supplementary Figure S18

We applied ChromDMM, ChIP-partitioning and SPar-K with the flip and shift state inference to cluster ENCODE data containing 10 chromatin features extracted at enhancer regions. For the details of the data, preprocessing and the definition of the enhancers, see Supplementary Section S4.2 and Osmala and Lähdesmäki (2020). Based on the ChromDMM fit, the enhancers were assigned to the most probably clusters and their profiles were re-aligned based on the inferred shift and flip states, for example for visualization. As a result, ChromDMM separated enhancers into six clusters, each with distinctive and refined combinations of chromatin feature patterns. Three of the six clusters are visualized as heatmaps and aggregated patterns in Fig. 4 (the full set of clusters and chromatin features are presented in Supplementary Fig. S18). In contrast, ChIP-partitioning and SPar-K failed to identify distinctive patterns and to refine the profile alignment and strand-orientation (Supplementary Figs. S20 and S21).

The ChromDMM enhancer clusters possess characteristic combinations of chromatin feature pattern shapes, spacings and signal strengths. The first cluster has symmetric and high enrichment of histone modification and MNase-seq signals with a steep decline of the signals in the middle of the profiles, indicating a nucleosome-free region. In addition, the nucleosome-free region is surrounded by a regular array of well-positioned nucleosomes. In contrast, in the clusters 2, 3 and 4, the nucleosome-free region and the well-positioning of the nucleosomes are obscured compared with the other clusters. Thus, the enhancers in these clusters may possess closed chromatin or mobile nucleosomes. The clusters 4–6 have asymmetricity in histone modification enrichment (clusters 4 and 5), in nucleosome positioning (clusters 5 and 6) and in RNA POL II occupancy (clusters 4 and 5). The asymmetricity in the RNA POL II ChIP-seq signal may reflect the direction of transcription. In addition, in the asymmetric clusters, the histone modifications are enriched on either of the two nucleosomes immediately flanking the anchor position (cluster 5) or spread widely (clusters 4 and 6).

#### 3.2.2 Biological validation of the inferred clusters

**Fig. 5.**
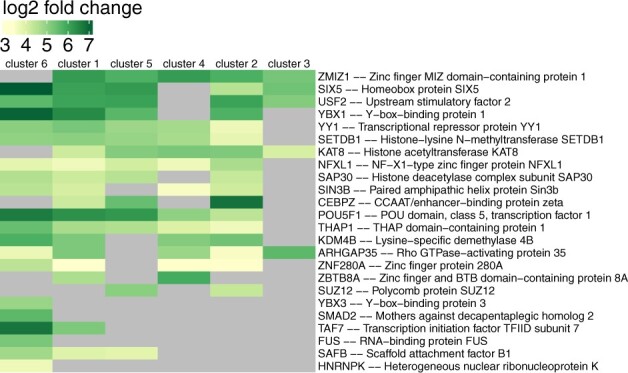
The fold enrichment of TRFs at the enhancer clusters identified by ChromDMM. The fold enrichments corresponding to *q*-value larger than 0.01 were masked out from the heatmap. The enrichment results for all 220 TRFs are presented in Supplementary Fig. S19

The enhancer clusters revealed by ChromDMM, ChIP-partitioning and SPar-K were investigated for the enrichment of the binding sites of transcription factors (TFs) and other regulatory proteins, collectively referred to as TRFs. The ChIP-seq peaks for 220 TRFs were downloaded from ENCODE. For each TRF-cluster pair, a significance test for the enrichment of a given TRF at the cluster was performed by the GAT tool (Heger *et al.*, 2013). A large majority of the enrichments were significant according to the *q*-value threshold 0.01. To reveal differences in the TRF enrichment between clusters, the fold enrichments were visualized as a heatmap, where the enrichments corresponding to *q*-value larger than 0.01 were masked out (see Supplementary Fig. S19 for ChromDMM clusters). The fold enrichments for TRFs which were significantly enriched in at least one ChromDMM cluster and simultaneously not enriched in at least one another cluster are presented in Fig. 5. For more details, see Supplementary Method S4.3.

The enrichment of TRFs in ChromDMM enhancer clusters reveals the potential biological significance of the distinctive chromatin feature patterns. Clusters 3 and 4 with obscured nucleosome-free regions and nucleosome positioning have less enrichment of TRFs than the four other clusters. In contrast, the first cluster with symmetric and strong signals has an enrichment for a large number of TRFs. Similarly to cluster 1, cluster 5 with strong asymmetry in the histone modification and RNA POL II signals has a high TRF enrichment. Asymmetric cluster 6 with strong average H3K27ac, H3K9ac and DNase-seq signals differs from the other clusters with unique enrichment for RNA binding and processing-related proteins (HNRNPK, FUS) and TFs SMAD2 and YBX3. In addition, clusters 1 and 6 have enrichment for the largest component and core scaffold of the TFIID basal TF complex (TAF7). Moreover, clusters 1, 5 and 6 are enriched for Scaffold attachment factor B1 (SAFB), a protein that binds DNA regions that are bound to the nuclear scaffold. Interestingly, SAFB may be involved in attaching the base of the chromatin loops to the nuclear scaffold and serving as a molecular base to assemble a transcriptosome complex in the vicinity of the actively transcribed genes (Nayler *et al.*, 1998). For comparison, the TRF enrichments at ChIP-partitioning and SPar-K clusters are visualized in Supplementary Fig. S22 and S23.

## 4 Conclusions

Exploring epigenetic datasets provides crucial information on key biological mechanisms such as gene regulation. An example of such data mining is the clustering of epigenomic signals and other chromatin features at regulatory elements, such as enhancers, to reveal the combinations of chromatin features with varying signal magnitudes and profile shapes. To appropriately account for the sparse, discrete, heterogeneous and overdispersed nature of the chromatin-feature data, probabilistic clustering methods have been developed.

We have proposed ChromDMM, a product Dirichlet-multinomial mixture model that provides a probabilistic method to cluster multiple chromatin-feature coverage signals extracted from the same locus. By employing simulated data, we demonstrated that the accuracy of ChromDMM increases with the increasing number of chromatin features. This indicates the need for a principled approach that considers the multiple chromatin features simultaneously when clustering regulatory elements. Moreover, we demonstrated that ChromDMM outperforms the previous methods ChIP-partitioning and SPar-K in clustering accuracy, particularly when the chromatin-feature coverages are low. In addition, ChromDMM learns the shift and flip states more accurately compared with ChIP-partitioning and SPar-K. The accuracy of ChromDMM to infer the clusters and shift states is further improved by mixture component parameter regularization and an informative shift state prior. Finally, we confirmed that BIC and AIC can detect the correct number of clusters.

We illustrated that ChromDMM identifies clusters with distinct epigenetic patterns when applied to ENCODE data containing 10 chromatin features quantified at enhancers. Moreover, the identified clusters are enriched for different sets of TRFs, suggesting that the clusters may vary in their biological characteristics. ChromDMM may therefore be a valuable method to reveal potential functionally distinct subclasses of regulatory elements.

## Supplementary Material

btac444_Supplementary_Data
